# Small RAB GTPases Regulate Multiple Steps of Mitosis

**DOI:** 10.3389/fcell.2016.00002

**Published:** 2016-02-17

**Authors:** Stéphanie Miserey-Lenkei, María I. Colombo

**Affiliations:** ^1^Institut Curie, PSL Research University, Molecular Mechanisms of Intracellular Transport Group, CNRS UMR 144Paris, France; ^2^Laboratorio de Biología Celular y Molecular, Instituto de Histología y Embriología-CONICET, Facultad de Ciencias Médicas, Universidad Nacional de CuyoMendoza, Argentina

**Keywords:** RABs GTPases, mitosis, endosomes, golgi complex, trafficking

## Abstract

GTPases of the RAB family are key regulators of multiple steps of membrane trafficking. Several members of the RAB GTPase family have been implicated in mitotic progression. In this review, we will first focus on the function of endosome-associated RAB GTPases reported in early steps of mitosis, spindle pole maturation, and during cytokinesis. Second, we will discuss the role of Golgi-associated RAB GTPases at the metaphase/anaphase transition and during cytokinesis.

## Introduction

In mammalian cells, GTPases of the RAB family are key regulators of multiple steps of membrane traffic. RAB GTPases play a central role in the formation of transport carriers from a donor membrane, movement of these carriers along cytoskeletal tracks and finally anchoring/fusion to the correct acceptor membrane (Stenmark, 2009). RAB GTPases represent a large family of small guanosine triphosphate (GTP)-binding proteins that comprise more than 60 known members. RAB GTPases are localized on distinct membrane-bound compartments and cycle between an active GTP-bound form and an inactive guanosine diphosphate (GDP)-bound form. The active GTP-bound forms bind to specific effectors and are potent activators of intracellular signaling networks. GDP-GTP cycling is regulated by guanine nucleotide exchange factors (GEFs). GTP-GDP cycling is regulated by GTPase-activating protein (GAPs) (Stenmark, 2009).

When cells enter mitosis, intracellular transport is arrested and intracellular compartments are disassembled and/or fragmented. This ensures an equal partitioning of organelles between daughter cells.

The first evidence that membrane trafficking events, and specifically secretion, are required for cytokinesis came from studies performed in plant cells (Jürgens, 2005; Van Damme et al., 2008). The contribution of membrane traffic for mitotic progression in eukaryotic cells was highlighted 15 years ago (Skop et al., 2001; Guertin et al., 2002; Echard et al., 2004; Schweitzer and D'souza-Schorey, 2004). Indeed, the first role for a RAB GTPase during cytokinesis was reported in *C. elegans* (Skop et al., 2001). As will be discussed in this review, since then, the role of several RAB GTPases has been extensively described at all stages of mitosis (Table 1). However, except few examples, the precise role played by RAB GTPases remains unknown. Indeed, a clear function has only been assigned to RAB11-, RAB35-, and RAB21-associated vesicles shown to transport specific signaling molecules at the cleavage furrow to allow progression through cytokinesis and exit of mitosis.

**Table 1:** **Summary of the described localization, phenotype, and function of endosomal- and Golgi associated RABs, at early stage of mitosis, metaphase/anaphase transition, and cytokinesis**.

**Stage of mitosis**	**RAB GTPase**	**Localization**	**Phenotype/described function if any**
Early Mitosis	5	In *Drosophila*: endosomes organized around the spindle poles (Capalbo et al., 2011)	In *Drosophila*: improper chromosome alignment before anaphase *via* its association with nuclear Lamin and Mud (Capalbo et al., 2011)
	5	In mammalian cells: clusters around spindle poles at the onset of mitosis (Serio et al., 2011)	In mammalian cells: defects in chromosome congression and marked prometaphase delay. Reduced localization of CENP-F to kinetochores (Serio et al., 2011)
	11	n. d.	In *C. elegans*: in association with dynein, regulation of aster-microtubule size, spindle alignment, and morphology of endoplasmic reticulum (Zhang et al., 2008)
	11	Motile structures organized around mitotic spindle and mitotic spindle poles (Hehnly and Doxsey, 2014)	In mammalian cells: disruption of astral microtubules, delayed mitosis, redistribution of spindle pole proteins. In association with dynein, spindle pole organization (Hehnly and Doxsey, 2014)
Metaphase/anaphase transition	6	Vesicles in the cytosol and cytosolic pool (Miserey-Lenkei et al., 2006)	Block at the metaphase/anaphase transition. In association with GAPCenA and p150*^*Glued*^*, transport of Mad2 from kinetochores to the spindle poles, leading to the inactivation of the Mad2 spindle checkpoint (Miserey-Lenkei et al., 2006)
	24	Mitotic spindle (Militello et al., 2013)	Misaligned metaphase chromosomes with abnormal spindle formation (Militello et al., 2013)
Cytokinesis	11	Vesicles accumulated around the cleavage furrow (Horgan et al., 2004; Wilson et al., 2005)	In *C. elegans* and mammalian cells: abnormal abscission. Brings membrane and signaling components required for successfull cytokinesis to the cleavage furrow (Skop et al., 2001; Horgan et al., 2004; Wilson et al., 2005; Schiel et al., 2011, 2012)
	35	Vesicles accumulated around the cleavage furrow (Dambournet et al., 2011)	Late abscission defects. Delivers OCRL to the intercellular bridge. OCRL regulates PtdIns(4,5)P2 hydrolysis and locally remodels F-actin cytoskeleton (Dambournet et al., 2011)
	21	Vesicles at the opposite poles of the daughter cells and at the cleavage furrow (Pellinen et al., 2008)	Multinucleated cells. Targeted trafficking of integrins to the cleavage furrow (Pellinen et al., 2008)
	8A	Midbody (Kaplan and Reiner, 2011)	Increased multinucleated cells. RAB8A, via DCDC5 and cytoplasmic dynein is transported to the cleavage furrow to coordinate late cytokinesis (Kaplan and Reiner, 2011)
	24	Midbody (Militello et al., 2013)	Multinucleated cells. Cytokinesis failure (Militello et al., 2013)
	6	n. d.	Cytokinesis failure (Bardin et al., 2015)

*N.d., Not determined*.

## Role of endosome-associated RABs

### The fate of endosomes during mitosis

At the entry of mitosis, early endosomes, recycling endosomes, and lysosomes disperse in the cytoplasm. It has been described that recycling endosomes are concentrated around the two poles of the mitotic spindle and at the extremities of the central spindle (Dunster et al., 2002; Schweitzer et al., 2005; Boucrot and Kirchhausen, 2007). In early mitosis, RAB5-positive early endosomes are found organized around the spindle poles (Capalbo et al., 2011; Serio et al., 2011; Lanzetti, 2012). During cytokinesis, an important endosomal trafficking takes place in proximity to the midbody (detailed below).

### Role of endosomal RABs during early steps of mitosis

#### Formation and positioning of the mitotic spindle

Successful cell division is dependent on the proper formation and precise positioning of the mitotic spindle. The assembly of the mitotic spindle starts in prophase with the nucleation of the microtubules by the centrosomes. Then, aster microtubules grow and extend toward the cell cortex (Lu and Johnston, 2013). In the case of symmetric division, as mitosis progresses, cortical polarity cues position the mitotic spindle in order that the cleavage furrow will bisect the cell in the middle of the central spindle in two equal parts during cytokinesis.

#### RAB11- and RAB5-positive endosomes in chromosome congression and organization of the mitotic spindle

**Figure 1:**
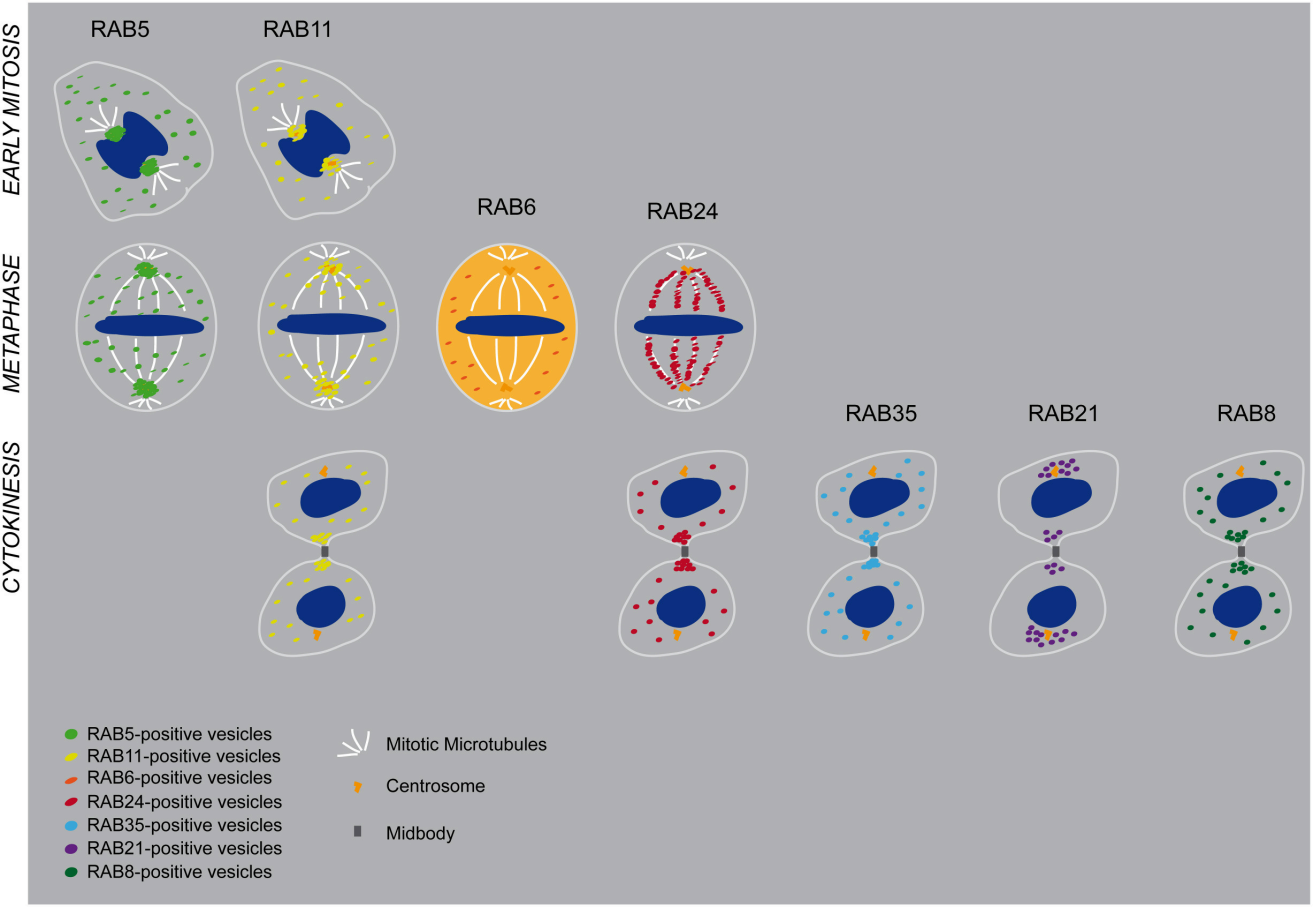
**This schematic illustrated partially Table 1**. Described localization of RAB5, RAB11, RAB6, RAB24, RAB35, RAB21, and RAB8A at early stage of mitosis, metaphase, and cytokinesis. In the case of RAB6 an active cytosolic pool has been reported.

In interphase, RAB11 is associated to recycling endosomes (Grant and Donaldson, 2009). At early stages of mitosis in *C. elegans*, RAB11, in association with dynein, regulates astral microtubule size, spindle alignment, and the morphology of endoplasmic reticulum (Zhang et al., 2008; Ai and Skop, 2009). The detailed localization of RAB11 in *C. elegans* at early stages of mitosis has not been described yet. In mammalian cells, a role for RAB11-positive endosomes in spindle pole organization and orientation was recently reported (Hehnly and Doxsey, 2014). Using time-lapse imaging, it was shown that RAB11 endosomes are found localized on the mitotic spindle and at the mitotic spindle poles (Hehnly and Doxsey, 2014; Table 1 and Figure 1). These mitotic recycling endosomes bind to microtubule-nucleating components and to dynein. Astral microtubule disruption, a mitotic delay and a redistribution of spindle poles proteins are observed following RAB11 inhibition. As proposed by the authors, RAB11 endosomes could be part of a dynein-dependent retrograde transport pathway bringing microtubule nucleating factors and spindle pole proteins to mitotic spindle poles (Das et al., 2015).

In interphase, RAB5 is associated with early endosomes. Two studies performed in *Drosophila* and mammalian cells reported the involvement of RAB5 at an early stage of cell division, where RAB5 acts by modulating the congression and segregation of chromosomes (Capalbo et al., 2011; Serio et al., 2011; Lanzetti, 2012). This function of RAB5 is evolutionary conserved. In both *Drosophila* and mammalian cells, at early mitosis, RAB5 localizes to endosomes organized around the spindle poles (Capalbo et al., 2011; Serio et al., 2011; Lanzetti, 2012; Table 1 and Figure 1). During *Drosophila* mitosis, RAB5 is required for proper chromosome alignment before anaphase (Capalbo et al., 2011). RAB5 associates *in vivo* with nuclear lamin and Mushroom Body Defect (Mud), the *Drosophila* homolog of the nuclear mitotic apparatus protein (NuMA), which is known to be important for spindle formation and maintenance in mammalian cells. RAB5 is required for the disassembly of the nuclear envelope at mitotic entry and the accumulation of Mud at the spindle poles (Capalbo et al., 2011). In mammalian U2OS cells, RAB5 silencing causes defects in chromosome congression and a marked prometaphase delay, due to a reduction in the localization of the protein CENP-F to kinetochores (Serio et al., 2011). CENP-F is a centromere-associated protein that contributes to the establishment of kinetochore-microtubule interactions. In mitosis, RAB5 can form a complex with CENP-F and regulates the accumulation of CENP-F to kinetochores through the regulation of its kinetic release from kinetochores (Serio et al., 2011). How RAB5 regulates the accumulation of CENP-F to kinetochores remains to be elucidated. Which population of RAB5 is active? Cytosolic or membrane-associated? The accumulation of CENP-F is done *via* vesicular transport or through the association with a common CENP-F/RAB5 effector? What are the effectors of RAB5 involved in chromosome congression? The identification of specific RAB5 effectors, using proteomic analysis for example, at early mitosis could help resolve these questions.

The other interesting point to address is the existence of RAB5-RAB11 endosomal maturation from prophase to telophase. In interphase cells, RAB5-labeled early endosomes can mature into RAB11-labeled late endosomes (Scott et al., 2014). As mitosis progresses, the amount of RAB5-positive endosomes decreases (Serio et al., 2011; Lanzetti, 2012) while RAB11-positive endosomes accumulate (Hobdy-Henderson et al., 2003).

#### RAB24 in mitosis

RAB24 is a member of the RAB GTPase family whose specific function is presently unknown. This atypical RAB is expressed ubiquitously but presents peculiar properties such as a very low intrinsic GTPase activity and inefficient prenylation in comparison with other RABs (Erdman et al., 2000). The description of the localization of RAB24 in interphase and mitosis is mostly based on overexpression of tagged constructs although the distribution of the endogenous protein has also been analyzed by using specific antibodies.

In interphase, RAB24 presents a broad intracellular localization: a perinuclear reticular localization frequently surrounding the nucleus (Olkkonen et al., 1993; Erdman et al., 2000; Munafó and Colombo, 2002), nuclear inclusions (in the case of an “empty mutant”) (Maltese et al., 2002; Wu et al., 2006), a co-localization with ER-Golgi intermediate compartments and the cis-Golgi marker CTR433 (Munafó and Colombo, 2002), a partial overlap with late endosomal markers such as RAB7 (Olkkonen et al., 1993) and finally, after stimulation of autophagy, an association to autophagosomes (Munafó and Colombo, 2002).

During mitosis, RAB24 distribution shows a distinct pattern depending on the stage of mitosis (Militello et al., 2013; Table 1 and Figure 1). Throughout metaphase and anaphase, RAB24 is present at the mitotic spindle. In metaphase, Rab24 overexpression causes chromosomes misalignment with abnormal spindle formation. In addition, a partial overlap of RAB24 with tubulin has also been observed. RAB24's association with microtubules was also demonstrated both *in vivo* and *in vitro* (Militello et al., 2013). Thus, several mitotic steps are modulated by RAB24, perhaps *via* its interaction with microtubules.

However, how RAB24 can associate directly to tubulin has to be elucidated. Is the active RAB24 fraction membrane-associated or cytosolic? Until now, two common RAB24/RAB6 effectors, R6IP1 and GAPCenA have been identified (see detailed discussion below). The identification of specific RAB24 effectors involved in early stages of mitosis would be critical to determine the precise role of this protein at the molecular level.

#### The unknown function of RAB4 in mitosis

The functions of many RABs require continuous association and dissociation cytoplasm-membrane cycles. In mitosis, this cycle is disrupted in the case of RAB4. The phosphorylation of RAB4 by the mitotic kinase p34^cdc2^ (Bailly et al., 1991) increases the amount of RAB4:GTP in the cytoplasm. This phosphorylation likely results in a less efficient recruitment of RAB4 effectors onto mitotic endosomal membranes and arrest of the endocytic process (Bailly et al., 1991; van der Sluijs et al., 1992; Gerez et al., 2000). RAB4:GTP is maintained in the cytosol through an association with the peptidyl–prolyl isomerase Pin1 (Gerez et al., 2000). The precise function of RAB4 during mitosis remains unknown. The possible phosphorylation of other RABs is discussed in the last section.

### Role of endosome-associated RABs during cytokinesis

#### Cytokinesis

Cytokinesis is the terminal stage of eukaryotic cell division. At this stage, in the case of symmetric division, the cytoplasm of the dividing cell is partitioned equally between two daughter cells. Cytokinesis involves complex changes in cell shape. A narrowing acto-myosin contractile ring is formed between the poles of the mitotic spindle and is responsible for ingression of the cleavage furrow (McCollum, 2004). However, this is not the only mechanism that drives cytokinesis. Membrane trafficking is also crucial for abscission (Matheson et al., 2005; Barr and Grüneberg, 2007). Indeed, endosomes constitute a reservoir of new membrane which are incorporated into the cleavage furrow (Boucrot and Kirchhausen, 2007). In addition, a specific endosome-dependent targeting of key proteins involved in the final stage of cytokinesis, implicating RAB11 and RAB35, has also been reported. This may explain why numerous endosome-associated RABs have been implicated in cytokinesis.

#### RAB11, RAB35, RAB21, RAB8A, and RAB24 in cytokinesis

In *C. elegans*, a role for RAB GTPases in late stages of cell division has been documented (Yu et al., 2007). siRNA-mediated depletion of RAB11 leads to cytokinesis defects, including furrow regression and abnormal abscission (Skop et al., 2001). Both RAB11 and its interacting protein RAB11-FIP3 localize to the cleavage furrow during cytokinesis (Horgan et al., 2004; Table 1 and Figure 1). In mammalian cells, RAB11 and RAB11-FIP3 containing recycling endosomes are found accumulated near the cleavage furrow (Wilson et al., 2005).

In interphase, RAB35 localizes to the endocytic recycling pathway, much like RAB11. RAB35 functions at early endosomes in a fast-recycling endocytic pathway prior to the slow recycling endosomal step regulated by RAB11. In cytokinesis, RAB35 is essential for post-furrowing stages (Kouranti et al., 2006). In interphase and mitosis, RAB35 binds to the phosphatase ocucerebrorenal syndrome of Lowe (OCRL). During the post-furrowing cytokinesis stages, OCRL is targeted to the cleavage furrow via RAB35-positive endosomes (Table 1 and Figure 1). There, OCRL regulates PtdIns(4,5)P2 hydrolysis and locally remodels the F-actin cytoskeleton at the intercellular bridge. This event is important for normal cytokinesis abscission (Dambournet et al., 2011). In addition, an ARF6/RAB35 cascade controlling endocytic recycling and successful cytokinesis has been described (Chesneau et al., 2012). EPI64B (a GAP for RAB35) acts as an effector of Arf6 and negatively regulates RAB35 activation. This molecular mechanism controls the RAB35 pathway, including RAB35 localization at the bridge and hence completion of cytokinesis (Chesneau et al., 2012). Regarding the signaling function of RAB11-positive endosomes during cytokinesis, it has been shown that FIP3/RAB11/Arf6 endosomes deliver key proteins involved in late abscission steps, SCAMP2/3 and p50Rho-GAP, to the cleavage furrow (Schiel et al., 2012).

In interphase, RAB21 is an endosomal RAB involved in the regulation of cell adhesion and migration. RAB21 acts through the targeted trafficking of integrins *via* its association with integrin alpha tails (Pellinen et al., 2006). In cytokinesis, RAB21 targets integrins to the cleavage furrow (Pellinen et al., 2008). In telophase and cytokinesis, RAB21 vesicles are localized to the opposite poles of the daughter cells and to the cleavage furrow (Pellinen et al., 2008). As suggested by Pellinen et al. (2008), these two pools of RAB21 vesicles would have two different functions. The pool of RAB21 vesicles localized to the opposite poles of the daughter cells are associated with β1-intergrin and are forming protrusions at the level of the matrix. These structures would help the mechanical separation of the two daughter cells. The pool of RAB21 vesicles targeted at the cleavage furrow would regulate RhoA activity and consequently the activity of known RhoA effectors. Loss of RAB21 gene expression in human cancer leads to the accumulation of multinucleated cells (Pellinen et al., 2008). In addition, abnormal integrin trafficking was linked with the generation of aneuploidy and cell transformation. In human prostate and ovarian cancer samples, increased malignancy is correlated with downregulation of RAB21. Long-term depletion of RAB21 is sufficient to induce chromosome number aberrations in normal human epithelial cells (Högnäs et al., 2011).

RAB8A has also been implicated in cytokinesis (Table 1, Figure 1). The protein doublecortin domain-containing protein 5 (DCDC5) interacts with cytoplasmic dynein and RAB8 (RAB8A), as well as with the RAB8 nucleotide exchange factor RABin8 (Kaplan and Reiner, 2011). Following DCDC5 knockdown, the duration of metaphase/anaphase transition and cytokinesis, as well as the amount of multinucleated cells, increases. DCDC5 therefore appears to play a role in mediating dynein-dependent transport of RAB8-positive vesicles to coordinate late cytokinesis events.

In cytokinesis, RAB24 is found associated to the midbody (Table 1, Figure 1). In cells overexpressing RAB24 or in RAB24 silenced cells, long chromatin bridges connect cells undergoing cell division (Militello et al., 2013). As a consequence, cells are unable to undergo abscission and cytokinetic furrows eventually retract, leading to the appearance of binucleated and multinucleated cells. It is likely that the observed defects in cell division are due to defects in congression and segregation of chromosomes as explained in the previous section.

## Role of Golgi-associated RABs

### Fate of the Golgi in mitosis

In mitosis, the Golgi complex is dispersed in regulated steps into tubular–reticular and vesicular elements (Misteli and Warren, 1995; reviewed in Jongsma et al., 2015). Post-mitotic Golgi reassembly begins in telophase (Gaietta et al., 2006), and several papers have suggested the existence of a “Golgi mitotic checkpoint” which monitors the inheritance of the Golgi complex (Sütterlin et al., 2002; Hidalgo Carcedo et al., 2004; Colanzi and Corda, 2007; Colanzi et al., 2007). The intermediate compartment has recently been proposed to be implicated in Golgi inheritance (Marie et al., 2013).

### The Golgi-associated RAB6 at the metaphase/anaphase transition

RAB6 is associated with Golgi and *trans*-Golgi (TGN) membranes in interphase and is a key regulator of Golgi homeostasis (Grigoriev et al., 2007; Miserey-Lenkei et al., 2010; Goud and Akmanova, 2012). Two RAB6 isoforms, termed RAB6A and RAB6A', are expressed in mammalian cells (Echard et al., 2000). Several of the previously identified RAB6-interacting proteins have been shown to function during mitosis. The RAB6 GTPase-activating protein (GAP) termed GAPCenA, is partially localized to the centrosome in interphase (Cuif et al., 1999). The kinesin-like protein RABkinesin-6 (also named RAB6KIFL, MKlp2, KIF20) (Echard, 1998), which expression is upregulated at the onset of mitosis (Hill et al., 2000; Fontijn et al., 2001), is involved in the localization of Polo-like kinase 1 (Plk1), Aurora B and Cdc14A at the central spindle (Neef, 2003; Gruneberg, 2004). These proteins are required for successful cytokinesis. In addition, RAB6A and RAB6A′ also interact directly with p150^*Glued*^, a subunit of the dynactin complex (Short et al., 2002). The dynein/dynactin complex is involved in many aspects of mitosis and specifically in the transport of checkpoint proteins such as Mad2 away from kinetochores at the metaphase/anaphase transition (Echeverri et al., 1996; Merdes et al., 2000; Howell et al., 2001; Wojcik et al., 2001; Basto et al., 2004; Siller et al., 2005). The Mad2-spindle checkpoint senses an absence of tension of mono-oriented chromosomes and defects in kinetochore attachment (Biggins and Murray, 2001; Musacchio and Hardwick, 2002).

During mitosis, RAB6 is associated with intracellular vesicles (Table 1, Figure 1). A cytosolic active pool of RAB6 has also been highlighted (Miserey-Lenkei et al., 2006). When the RAB6A' isoform or GAPCenA functions are inhibited, cells are unable to progress through the metaphase/anaphase transition normally. Such cells are blocked in metaphase despite displaying a normal Golgi fragmentation and activation of the Mad2-spindle checkpoint (Miserey-Lenkei et al., 2006). Furthermore, the RAB6 effector p150^*Glued*^ remains associated with some kinetochores. The function of RAB6A' is thus required for the dynamics of the dynein/dynactin complex at the kinetochores and consequently the inactivation of the Mad2-spindle checkpoint.

The observation that the cytosolic pool of RAB6 appears to be in its GTP-bound conformation during mitosis was surprising. Indeed active forms of RABs are supposed to be membrane-bound (Zerial and McBride, 2001; Pfeffer and Aivazian, 2004). However, RAB4 is also found in its GTP-bound form without membrane association during mitosis (Gerez et al., 2000). It has been shown that RAB4:GTP is maintained in the cytoplasm via its association with the peptidyl–prolyl isomerase Pin1 (Gerez et al., 2000). It remains to be established whether RAB6 is phosphorylated during mitosis and also interacts with Pin1 or to another protein that fulfills a similar function.

### RAB6 during cytokinesis

The laboratory of B. Goud has recently generated mice with a conditional null allele of RAB6A (Bardin et al., 2015). Time-lapse videomicroscopy experiments performed on RAB6 KO MEFs display two interesting phenotypes: a defect in mitosis, as previously reported in HeLa cells (Miserey-Lenkei et al., 2006) and a cytokinesis failure (Bardin et al., 2015). Such a role for RAB6 has not been previously observed. Several RAB6 partners have been implicated in cytokinesis and could explain how RAB6 functions during cytokinesis. Cells depleted of RAB6IP1, a RAB6 effector that also interacts with RAB11, display cytokinesis defects (Miserey-Lenkei et al., 2007). RAB6 interacts in interphase (Miserey-Lenkei et al., 2010) with Myosin-II, a crucial regulator of cytokinesis (McCollum, 2004). RAB6 interacts with the kinesin KIF20A, known to play a critical role during mitosis (see above). It will be interesting to investigate how RAB6 coordinates the function of these different motors and its other partners during cytokinesis, and to identify which cargoes are transported by RAB6 to the interconnected bridge during cytokinesis.

#### The existence of a RAB6-RAB24 interplay at the metaphase/anaphase transition and in cytokinesis?

Interestingly, RAB24 and RAB6 disruption share a common phenotype: a block in mitosis at the metaphase/anaphase transition and two common effectors, GAPCenA and RAB6IP1. As discussed above, RAB6 and GAPCenA function are required for the metaphase/anaphase transition (Miserey-Lenkei et al., 2006). In the laboratory of M. Colombo, it has been shown that RAB24 interacts with GAPCenA and colocalizes with GAPCenA at the centrosome (Militello et al., 2013). In addition, depletion of R6IP1 leads to a block at the metaphase/anaphase transition and a defect in cytokinesis (Miserey-Lenkei et al., 2007). Preliminary results from the laboratory of M. Colombo indicate that in addition to RAB6 and RAB11, R6IP1 also interacts with RAB24. The existence of these common interactions, with GAPCenA and R6IP1, may in part explain the increased number of cells arrested in metaphase observed in RAB24-depleted cells (Militello et al., 2013). It would now be interesting to address the existence of a dialogue between RAB24, RAB6 in association with GAPCenA and R6IP1 to determine how the function of these proteins is coordinated at the metaphase/anaphase transition.

## Conclusions

Several members of the RAB GTPases are clearly key regulators of mitotic progression. However, the precise role of this family of proteins in mitosis is still poorly understood. Several questions remain to be addressed.

RAB4 is phosphorylated by the mitotic kinase p34^cdc2^ during mitosis (Bailly et al., 1991). This phosphorylation increases the amount of RAB4:GTP in the cytoplasm. Many key cell cycle proteins are regulated through phosphorylation by key mitotic kinases. Does phosphorylation also regulate the activity of RABs during mitosis? Phosphorylation could allow the appearance of a RAB population specific of mitosis and thus coordinate an interphase and mitotic function. Phosphorylated RABs would associate with specific mitotic effectors. In early mitotic stage, the existence of an active cytosolic pool has been demonstrated (RAB6) or is hypothesized (RAB5 with kinetochores, RAB24 with tubulin). Are RAB6, RAB5, and RAB24 phosphorylated at the entry of mitosis? Which kinases are involved?

The existence of a phosphorylated population specific of mitosis can also be addressed for RAB effectors. Are they phosphorylated to allow a specific relocation and association to defined mitotic partners? Another questions to address would be to investigate whether RABs effectors identified in mitosis are similar to the one involved in interphase and have similar functions.

RAB11 is playing a role in mitosis and cytokinesis. How are the two processes coordinated and regulated? Is it through RAB11 phosphorylation or through RAB11-interaction with specific effectors leading to its restricted relocation in its active form to specific areas? Is RAB11 phosphorylated at the entry of mitosis allowing the coordination of its interphase and mitotic function?

Finally, using different approaches, several studies have highlighted the role of new RABs in mitosis and cytokinesis (Kouranti et al., 2006; Neumann et al., 2010), in addition to the RABs discussed in this review, namely RAB37, RAB7, RAB22A, RAB25. Moreover, a transcriptional analysis approach has allowed the identification of RAB and RAB effector genes deregulated in bladder cancer (Ho et al., 2012). This study enabled the identification of several new RABs (RAB23, RAB20, RAB27) and some of their effectors. The next step is now to address the precise function of these new RABs in mitosis.

## Author contributions

All authors listed, have made substantial, direct and intellectual contribution to the work, and approved it for publication.

## Funding

This work was supported by the Centre National de la Recherche (CNRS), the Institut Curie and the Fondation ARC pour la Recherche Sur le Cancer.

### Conflict of interest statement

The authors declare that the research was conducted in the absence of any commercial or financial relationships that could be construed as a potential conflict of interest.
